# Monoclonal Antibodies against Small Molecule Natural Products and Their Applications, Eastern Blotting and Knockout Extract

**DOI:** 10.3390/ph4070950

**Published:** 2011-06-28

**Authors:** Yukihiro Shoyama

**Affiliations:** Faculty of Pharmaceutical Science, Nagasaki International University, 2825-7 Huis Ten Bosch, Sasebo, Nagasaki 859-3298, Japan; E-Mail: shoyama@niu.ac.jp; Tel.: +81-956-20-5653; Fax: +81-956-20-5653

**Keywords:** monoclonal antibody, natural product, ginseng saponin-ginsenoside, Eastern blotting, knockout extract

## Abstract

To determine the hapten number in hapten-carrier protein conjugate matrix-assisted laser desorption/ionization (MALDI) tof mass spectrometry was applied. Highly specific anti-ginsenoside Rb_1_ and Rg_1_ monoclonal antibodies (MAbs) were prepared. Ginsenosides were developed on thin layer chromatography (TLC) plates which were covered by a polyvinylidene difluoride (PVDF) membrane resulting in blotting. The membrane was treated with NaIO_4_ solution to release the aldehyde group on the sugar moiety of the ginsenosides. By treatment of the membrane with a protein solution the ginsenoside-protein conjugation as a Schiff-base occurred, which can function to fix it to the PVDF membrane. A part of the ginsenoside aglycone was reacted with anti-ginsenoside Rb_1_ MAb, secondary MAb conjugated with enzyme and finally a substrate was added, resulting in a specific and highly sensitive staining that we named Eastern blotting. Furthermore, it makes one-step isolation of ginsenoside Rb_1_ possible using an immuno-affinity column conjugated with anti-ginsenoside Rb_1_ MAb. Furthermore, immunoaffinity concentration was carried out allowing high sensitivity analysis of lower concentrations of ginsenoside Rb_1_ so that several unknown bands could be structurally determined.

## 1. Introduction

In the recent rapid development of the molecular biosciences and their biotechnological applications, and owing to their specific affinity, immunoassay systems using MAb against drugs and small molecular weight bioactive compounds have become an important tool for studies on receptor binding analysis, enzyme assay, and quantitative and/or qualitative analytical techniques in animals or plants. The immunoblotting method is based on the Western blotting technique that utilizes the antigen-antibody binding properties and provides a specific and sensitive detection of higher molecule analytes like peptides and proteins. Previously we prepared various kinds of MAb against natural products like forskolin [1], solamarigine [2], crocin [3], marihuana compounds [4], opium alkaloids [5], ginsenosides [6,7], berberine [8], sennosides [9], paeoniflorin [10], glycyrrhizin [11,12], ginkgolic acid [13], aconitine alkaloid [14], baicalin [15] and so on, and developed individual competitive enzyme-linked immunosorbent assays (ELISAs) as a high sensitive, specific, and simple methodology.

Since the confirmation of hapten number in synthesized antigens is most important in the first stage of MAb preparation, its determination method will be discussed first of all. As an application of MAb the MAb against ginsenosides have been prepared resulting in the development of a new technique that we have named the Eastern blotting method and the knockout extract preparation. They will be introduced in this paper.

## 2. Experimental

### 2.1. Determination of Hapten Number in Hapten-Carrier Protein Conjugates

Determination of hapten number in hapten-carrier protein conjugates was carried out by MALDI tof mass spectrometry. A small amount the antigen conjugate was mixed with an excess of sinapinic acid in an aqueous solution containing trifluoroacetic acid. The mixture was placed inside a MALDI tof mass monitor and irradiated with a N_2_ laser. The ions formed by each pulse were accelerated and detected [16-18].

### 2.2. Preparation of MAb against Ginsenoside Rb_1_

Preparation of MAb against ginsenoside Rb1 was done as follows: ginsenoside Rb1 containing sugars was treated by NaIO_4_ solution to cleave the sugar part and release aldehyde groups which can be combined with a carrier protein. The synthesized antigen was injected into mice and anti-ginsenoside MAb prepared by the standard method in my laboratory [7,19].

### 2.3. Eastern Blotting

Eastern blotting started with the development of components on a TLC plate. The developed TLC plate was covered by a PVDF membrane and a blotting solution added, then it was heated for a short period. The blotted PVDF membrane was treated with NaIO_4_ solution following the addition of BSA. The conjugated glycosides (ginsenosides) on the membrane were washed and treated with anti-ginsenoside Rb_1_ MAb and then peroxidase-labeled goat anti-mouse IgG MAb. Finally the PVDF membrane was exposed to substrate, 4-chloro-1-naphthol, and H_2_O_2_ solution. For staining by anti-ginsenoside Rg1 MAb, the blotted PVDF membrane was treated in the same way as anti-ginsenoside Rb_1_ MAb, except that it was exposed to 3-amino-9-ethylcarbazole and H_2_O_2_ solution [12].

### 2.4. Preparation of an Immunoaffinity Column Using Anti-Ginsenoside Rb_1_ MAb and Immunoaffinity Concentration

Purified anti-ginsenoside Rb1 MAb in diluted Bio-Rad Affi Gel Hz coupling buffer was dialyzed against the coupling buffer. NaIO_4_ solution was added to the anti-ginsenoside Rb_1_ MAb solution and stirred gently at room temperature in the dark. After the reaction, glycerol was added to the reaction mixture and stirred to inactivate NaIO4, and then dialyzed again. The Affi-Gel Hz Hydrazide gel was coupled with anti-ginsenoside Rb1 MAb treated by NaIO_4_ to give immunoaffinity gels which were packed into a plastic column [20].

### 2.5. Immunoaffinity Concentration and Preparation of Knockout Extract

The extracts of ginseng roots were loaded on the immunoaffinity column and allowed to stand overnight. The column was washed by a phosphate buffer as washing solution, and then eluted with 100 mM acetate buffer containing 0.5 M KSCN and 20% methanol. The washed solution (knockout extract) was deionized and concentrated for Eastern blotting and H_2_SO_4_ staining. The eluted solution was also deionized and concentrated for two stainings as described above [21].

## 3. Results and Discussion

### 3.1. Analytical Methodology for Determination of Hapten Number in Antigen, Hapten-Carrier Protein Conjugate

For production of MAb, synthesis of hapten which is derived from immune antigen and linker bridge, and carrier protein conjugates is necessary. There had been no direct and appropriate methods for the determination of haptens conjugated with carrier proteins without differential UV analysis, radiochemical or chemical methods. Therefore, immunization by the injection of hapten-carrier protein conjugate was unreliable. Wengatz *et al.* [22] determined the hapten density of immunoconjugates by matrix-assisted UV laser desorption/ionization mass spectrometry. We also reported the direct analytical method of hapten and carrier protein conjugates by a MALDI tof mass spectrometry using an internal standard.

**Figure 1:**
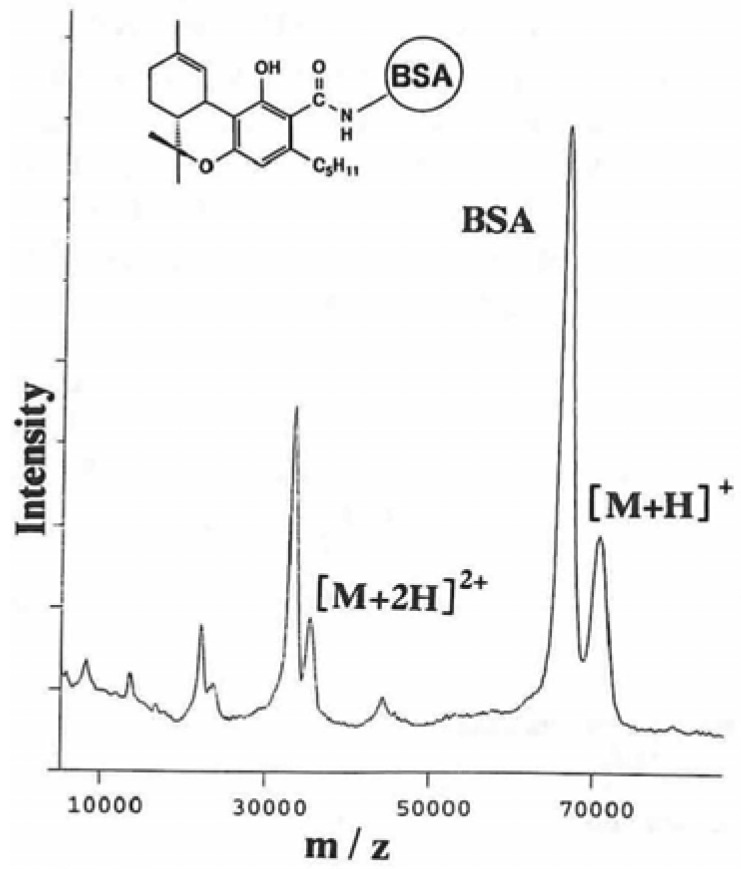
Matrix-assisted laser desorption/ionization tof mass spectrometry of tetrahydrocannabinolic acid-BSA conjugate.

Figure 1 shows the MALDI tof mass spectrum of tetrahydrocannabinolic acid (THCA)-BSA conjugate and BSA used as an internal standard. This shows only the singly, doubly and triply ionized molecule ions of the intact conjugate. The sharp peak at *m/z* 66,465 is the [M + H]^+^ peak of BSA. A small [M + H]^+^ peak of the THCA-BSA conjugate is at *m/z* 70,792, indicating that the calculated molecular mass of the THCA-BSA conjugate is 70,581 using a calculated molecular mass of 66,267 for BSA. The calculated molecule mass of the THCA moiety is 4,314. From this result, 12.7 molecules of THCA are combined with BSA [18]. This method is suitable for small molecule natural products including glycosides like ginsenosides.

### 3.2. Preparation of MAb against Natural Products

Many other methods have been employed in the determination of botanical constituents. They include spectral methods such as infrared (IR), nuclear magnetic resonance (NMR), and circular dichroism (CD), and other chromatographic methods such as ion chromatography (IC), capillary electrophoresis (CE), high-speed counter current chromatography (HSCCC) and so on. Compared to TLC, GLC and HPLC methods, the ELISA method was more sensitive and selective. Furthermore, no pretreatment of crude extracts is necessary. As an outstanding determination method, it is possible to study a large number of natural products. Since natural product extracts consist of various chemical constituents (for example licorice contains 470 components or more), in general, some pretreatment is necessary for HPLC and other chromatographic analysis methods. ELISA, however, can determine the concentration of components directly without any pretreatment. Therefore, ELISA was be used to measure the concentration of ginsenoside Rb_1_ in ginseng and traditional Chinese medicines (TCMs).

#### 3.2.1. Preparation of MAb against Ginsenosides and ELISA as an Assay System

Ginseng, the crude drug of *Panax ginseng*, is one of the most important natural medicines in many countries. It has been used to enhance stamina and capacity to cope with fatigue and physical stress, and as a tonic against cancers, disturbances of the central nervous system (memory, learning and behavior), hypothermia, carbohydrate and lipid metabolism, immune function, the cardiovascular system and radioprotection [23]. It contains more than 30 kinds of dammarane and oleanane saponins considered to be pharmacologically active components. Ginsenoside Rb_1_ is the main saponin in ginseng. However, since the concentration in the ginseng root or the root extract varies depending on the method of extraction, subsequent treatment, or even the season of its collection [24], standardization of quality is required. For this purpose we have prepared anti-ginsenoside Rb1 [6] and Rg1 MAbs [7]. The immunoassay system using MAb is not frequently used for naturally occurring smaller molecular weight bioactive compounds. Preparation of MAbs is difficult, but is one of the most important steps for the analysis of natural products. As a typical natural product, the preparation of MAb against the ginseng saponin ginsenoside Rb_1_ will be discussed.

A hybridoma producing MAb reactive to ginsenoside Rb1 was obtained by the general procedure and classified into IgG2b which had k light chains. The reactivity of IgG type MAb, 9G7 was tested by varying antibody concentration and by performing a dilution curve. The antibody concentration was selected for competitive ELISA. The free MAb following competition is bound to polystyrene microtiter plates precoated with ginsenoside Rb_1_-HAS. Under these coditions, the full measurement range of the assay extends from 20 to 400 ng/mL [6].

Cross-reactivity is the most important factor in determining the value of an antibody. Since the ELISA for ginsenoside Rb_1_ was established for phytochemical investigations involving crude plant extracts, the assay specificity was checked by determining the cross-reactivity of the MAb with various related compounds. The cross-reactivity data of MAb that was obtained were examined by competitive ELISA and calculated using picomole amounts of ginsenoside Rb_1_. The cross-reactivity of ginsenoside Rc and Rd, which possess a diglucose moiety attached to the C-3 hydroxy group, were weak compared to ginsenoside Rb1 (0.024 and 0.020%, respectively). Ginsenoside Re and Rg_1_ showed no cross-reactivity (less than 0.005%). It is evident that the MAb reacted only with a small number of structurally related ginsenoside Rb_1_ molecules, and very weakly, and did not react with other steroidal compounds like glycyrrhizin, gigitoxin, tigogenin, tigonin and solamargine.

In our ongoing studies on MAbs against ginseng saponins, anti-ginsenoside Rg_1_ MAb [7] and Re [25] have been prepared and their ELISA set up. Anti-ginsenoside Rg_1_ MAb was also highly specific like anti-ginsenoside Rb_1_. On the other hand, anti-ginsenoside Re MAb showed wide cross-reactivity. Therefore, the MAb can be used for the analysis for the total ginsenoside concentration.

### 3.3. Application of MAb in the Natural Products Field

Although Western blotting is a common assay methodology for high molecular weight substances, this method has not been employed for small molecules, as direct immunostaining of such compounds on a TLC plate is as yet unknown. Therefore, a new method for such small molecular compounds is required. Moreover, if small molecules can be blotted to a membrane, fixing them also requires a new methodology. Previously, we succeeded in separating small molecule compounds such as solasodine glycosides into a part of an epitope and fixing on the membrane [26], as follows.

#### 3.3.1. New Staining Method for Ginsenosides, “Eastern Blotting”

**Figure 2:**
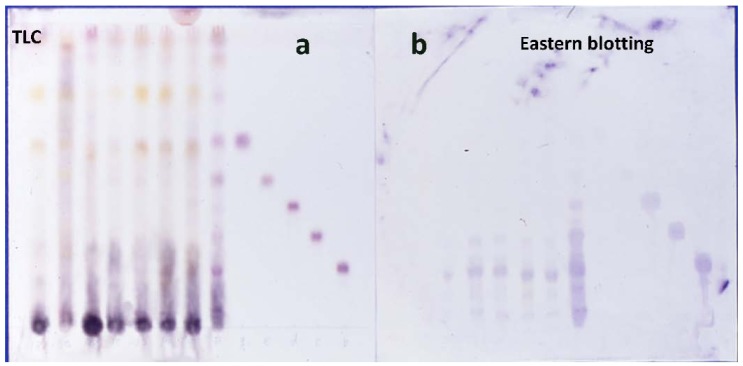
Eastern blotting of ginsenosides in traditional Chinese medicine formulas using anti-ginsenoside Rb1 MAb.

Figure 2 shows the H_2_SO_4_ staining and Eastern blotting of ginsenoside standards and TCM using anti-ginsenoside Rb_1_ MAb. It is impossible to determine the ginsenosides by TLC-staining by H_2_SO_4_ as indicated in Figure 2a. On the other hand, clear staining of ginsenoside Rb1 occurred by Eastern blotting, as indicated in Figure 2b. Furthermore, it became evident that Jigengtang and Dahuanggancaotang prescriptions that did not contain ginseng, as indicated by the absence of a ginsenoside Rb_1_ band. The Eastern blotting method was considerably more sensitive than that of H_2_SO_4_ staining. The H_2_SO_4_ staining detected all standard compounds. The Eastern blotting indicated only limited staining of ginsenoside Rb_1_, Rc and Rd, whose cross-reactivities were under 0.02% as shown in Figure 1b. We suggest that an aglycone, protopanaxadiol, and a part of the sugars may be of importance to the immunization and may function as an epitope for the structure of ginsenosides. In addition, it is suggested that the specific reactivity of sugar moieties in the ginsenoside molecule against anti-ginsenoside Rb_1_ MAb may be modified by the NaIO_4_ treatment of ginsenosides on the PVDF membrane, causing ginsenoside Rc and Rd to become detectable by Eastern blotting.

**Figure 3:**
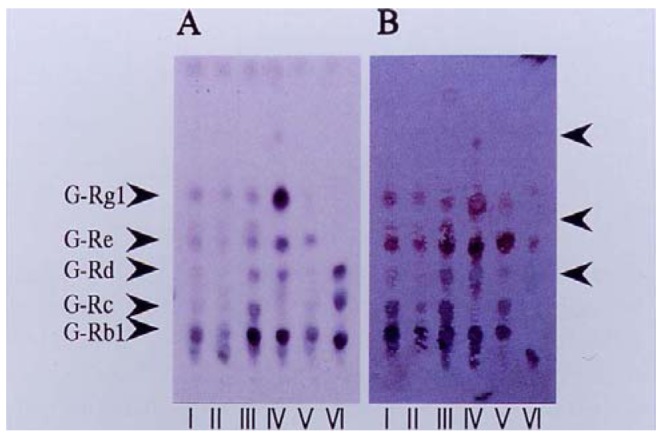
Double Eastern blotting staining of ginsenosides contained in various ginseng samples using anti-ginseenoside-Rb1 and anti-ginsenoside-Rg1 monoclonal antibodies.

When the mixture of anti-ginsenoside Rb_1_ and -Rg_1_ MAbs and the pair of substrates were tested for staining for ginsenosides, all ginsenosides, ginsenoside Rb_1_, -Rc, -Rd, -Re and -Rg_1_ were stained blue although the purple color staining for ginsenoside Rg_1_ was expected because 3-amino-9-ethylcarbazole and 4-chloro-1-naphotol might be different. Therefore, we performed successive staining of the membrane using anti-ginsenoside Rg_1_ and then anti-ginsenoside Rb_1_. Finally we performed the double staining of ginsenosides indicating that ginsenoside Rg1 and ginsenoside Re were stained purple and the other blue, as indicated in Figure 3. From this result, both antibodies can distinguish the individual aglycons protopanaxatriol and protopanaxadiol. For this application, the crude extract of various *Panax* species were analyzed by the newly developed double staining system. Major ginsenosides can be determined clearly by the double staining method, as indicated in Figure 3.

Therefore, it is possible to suggest that the staining color shows the pharmacological activity, inasmuch as the purple bands indicate ginsenosides which have protopanaxatriol as an aglycone and stimulation activity for the central nervous system (CNS). On the other hand, the blue color indicates gisenosides containing protopanaxadiol as an aglycone that possess a depression effect on the CNS. Moreover, the Rf value of ginsenosides roughly suggests the number of sugars attached to the aglycon. Both analyses make it possible to jointly identify which aglycone attaches and how many sugars it possesses, leading to the structure of the ginsenosides. In fact, three kinds of ginsenosides possessing protopanaxadiol-ginsenoside Rh_1_, -Rf, and 20-*O*-glucoginsenoside Rf in *P. ginseng* root were determined by coloring and Rf value by comparing them with the structures reported in the previous paper [27].

**Figure 4:**
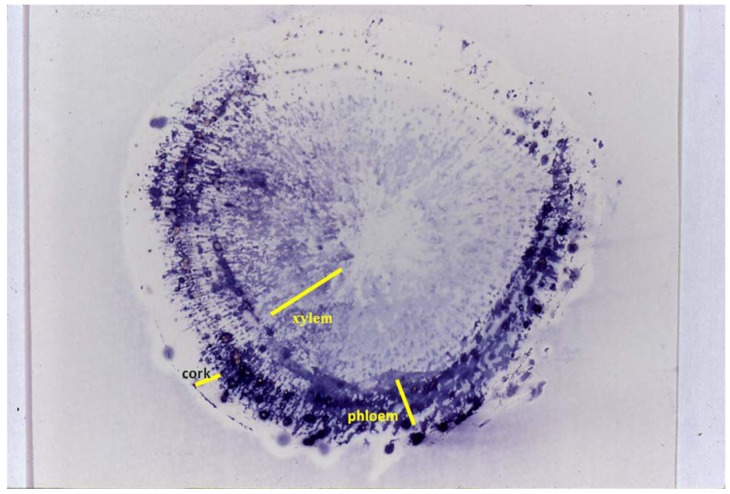
Immunocytolocalization of ginsenoside Rb1 in fresh *Panax ginseng* root using anti-ginsenoisde Rb1 Mab.

Figure 4 indicates immunolocalization of ginsenoside Rb_1_ in a ginseng root slice using anti-ginsenoside Rb1 MAb as another application of the Eastern blotting method. The phloem contained a higher concentration of ginsenosides than the xylem and cork part [28].

In the earlier experiments we carried out the blotted staining on PVDF membrane using MAb on solasodine glycosides and called it Western blotting [26]. Now we have applied this new methodology to licorice glycoside, glycyrrhizin and named it Eastern blotting [12] for studying ginsenosides [12], saikosaponin [29], and so on.

#### 3.3.2. Immunoaffinity Concentration and One Step Purification of Ginsenoside Rb_1_ by Immunoaffinity Column [21]

**Figure 5:**
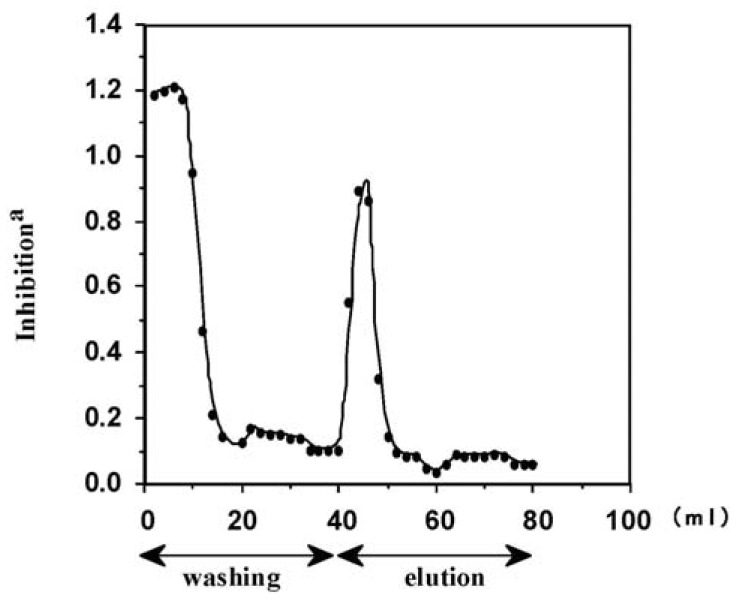
Elution profile of *Panax ginseng* crude extract on immunoaffinity column monitoring by ELISA using anti-ginsenoside-Rb1 MAb.

A crude extract of *P. ginseng* roots was loaded onto the immunoaffinity column and washed with the washing solution of phosphate buffer. Figure 5 shows the fraction 1-8 containing overloaded ginsenoside Rb_1_. The other ginsenosides Rg_1_, Rc, Re and Rd were also detected in these fractions by Eastern blotting (data not shown). A sharp peak appeared around fraction 20-24 eluted by acetate buffer containing KSCN and methanol to give pure ginsenoside Rb_1_.

Overloaded ginsenoside Rb1 was repeatedly immunoaffinity column choromatographed to separate ginsenoside Rb1 completely. The antibody was stable when exposed to the eluent, and the immunoaffinity column showed almost no decrease in capacity after repeated use more than 10 times under the same conditions, as was reported for a one-step separation of forskolin from a crude extract of *Coleus forskohlii* root [30]. This methodology is effective for the rapid and simple purification of ginsenoside Rb_1_ and may open up a wide field of comparable studies with other families of saponins for which an acceptable method for one-step separation has not yet been developed. Furthermore, to separate the total ginseng saponins, a wide cross-reactive MAb against ginsenoside like anti-ginsenoside Re MAb could be designed, as was done for the total solasodine glycosides by an immunoaffinity column using an anti-solamargine MAb [31]. A combination of immunoaffinity column chromatography, Eastern blotting and ELISA could be used to survey low concentrations of ginsenoside Rb1 of plant origin and/or in experimental animals and humans. In fact we have succeeded in the isolation of ginsenoside Rb_1_ from a different plant, *Kalopanax pictus* Nakai, which was not known previously to contain ginsenosides, using this combination of methods [32].

**Figure 6:**
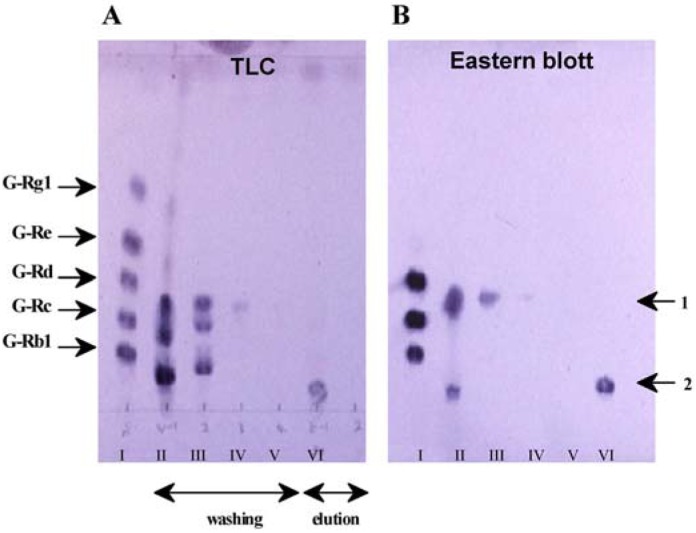
Purification and determination of ginsenosides of *P. japonicus* by immune-affinity column and Eastern blotting.

*P. japonicus* is distributed in Japan and China and is morphologically different from the other Panax species. Yahara *et al.* reported that no ginsenoside Rb_1_ was found in *P. japonicus* and isolated oleanane-type saponins called chikusetsusaponins and elucidated their structures [33]. Morita *et al.* examined the varieties of *P. japonicus* by chemical analysis of saponins. From these results, the concentration of ginsenoside Rb1 in this spp. might be only at trace levels [34]. However, we determined it by ELISA and found higher concentrations compared with previous reports, although approximately half the concentration of ginsenoside Rb1 was found by HPLC analysis. To clarify these differences, we used an immunoaffinity column for immunoaffinity concentration of ginsenoside Rb1. The crude root extract of *P. japonicus* was loaded on the immunoaffinity column and washed with the washing solvent and then with elution solvent, as already indicated. Figure 6 shows the H_2_SO_4_ staining (A) and the Eastern blotting (B) profiles of the two fractions separated by the immunoaffinity column. Fraction I eluted with the washing solvent showed many spots, indicating chikusetsusaponins, similar to the original extract of *P. japonicus*. However, fraction II contained a higher concentration of compound 1, although the other band was still detected on Eastern blotting. Compound 1 clearly indicated a dammarane saponin having protopanaxadiol as a framework and three sugars in a molecule compared to the Rf value of ginsenoside Rd, suggesting that compound 1 is chikusetsusaponin III. Finally, we identified compound 1 as chikusetsusaponin III by a direct comparison with authentic sample [35].

A clear unknown band of compound 2 appeared in fraction VI eluted with the elution solvent. Ginsenoside Rb1 was, however, not detected by Eastern blotting, although it was detected by TLC as indicated in Figure 6. It can be suggested that compound 2 has a molecular structure and cross-reactivity similar to those of ginsenoside Rb1 and seems to be related to the ginseng saponins having protopanaxadiol as an aglycone. Moreover, compound 2 might have the same sugar fragments and possess five sugar moieties in the molecule compared with ginsenoside Rb1, as indicated by their respective Rf values. From these pieces of evidence compound 2 might be chikusetsusaponin III-20-*O*-gentiobiose, chikusetsusaponin IV, which was identified by direct comparison with an authentic sample. Therefore, we concluded that *P. japonicus* did not contain ginsenoside Rb1, but did contain chikusetsusaponin IV, having the same aglycon and the same sugar components as ginsenoside Rb1 [35].

#### 3.3.3. Newly Established Knockout Extract for Ginsenosides

**Figure 7:**
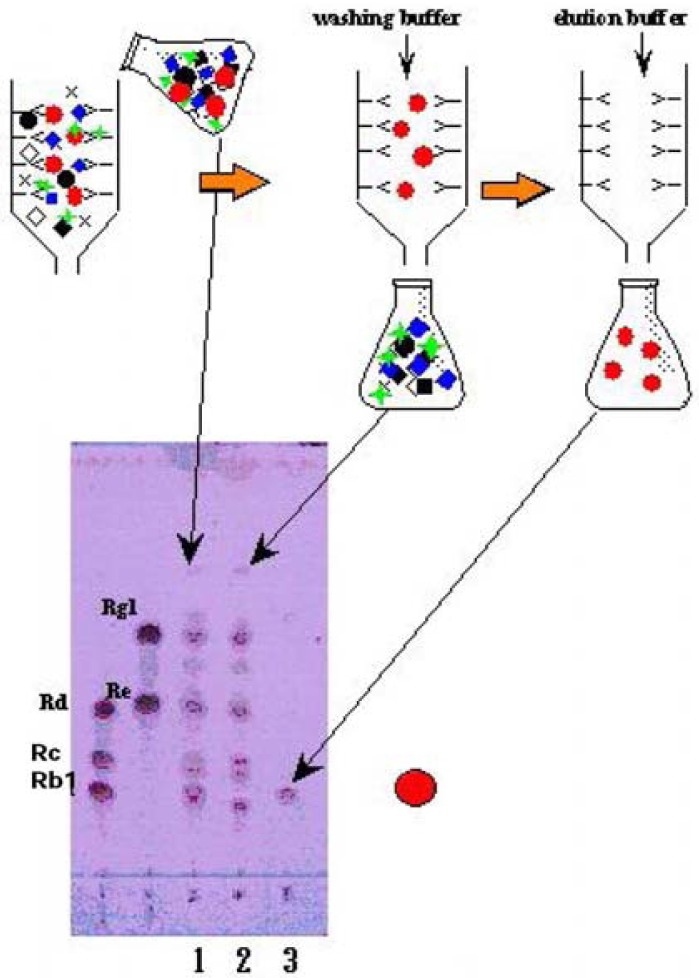
Preparation of knock-out extract eliminated ginsenoside-Rb1 from *Panax ginseng* crude extract using immunoaffinity column conjugated with anti-ginsenoside-Rb1 MAb.

Figure 7 shows the H_2_SO_4_ staining profile of TLC indicating the crude extract of ginseng root (lane 1), the collected deionized washing solution (lane 2) and the purified ginsenoside Rb_1_ (lane 3). Lane 2 indicated that this fraction contained all compounds except only ginsenoside Rb_1_ compared to that of crude extract. From this evidence we named this fraction the ginsenoside Rb_1_ knockout extract [21,36].

The knockout extract will be important for the confirmation of really bioactive components in crude extracts and/or TCMs.

## 4. Conclusions

Two unique applications using MAb, Eastern blotting and knockout extract have been introduced in this paper. The Eastern blotting method has great potential applications for the wide range of natural products, especially glycosides like the ginsenosides. When two kinds of MAbs can be used, the double staining resulted in a staining system that enhanced the separate staining of ginsenosides having protopanatriol or protopanaxadiol in a molecule. The staining color can be used to monitor the pharmacological activity suggesting that the purple spots containing protopanaxtriol as an aglycone indicate ginsenosides having CNS stimulatory activity [37]. On the other hand, the blue color indicates gisenosides having protopanaxadiol which possess a depression effect on the CNS [37]. Furthermore, the Rf value of ginsenosides suggests the number of sugars attached to the aglycon. Both evidences make it possible to confirm which aglycon is attached and how many sugars are combined with the aglycon.

A ginsenoside Rb_1_ knockout extract can be prepared using an immunoaffinity column conjugated with anti-ginsenoside Rb_1_ MAb. In this extract all compounds except only ginsenoside Rb_1_ are contained. This knockout extract may be able to support the pharmacological investigation for finding out a really active component in a crude drug and/or TCM.

## Figures and Tables

[M + H] indicates the molecular weight of the conjugate, from which the hapten number can be calculated.

Samples: 1) Jigengtang, 2) Dahuanggancaotang, 3) Nenshenyangyongtang, 4) Sijunzitang, 5) Nenshentang, 6) Buanxiaxiexintang, 7) Xiaocaihutang, 8) Crude ginseng extract. Lanes 1 and 2 do not contain ginseng. Standard of ginsenosides indicated, ginsenosidr-Rg1, -Re, -Rd, -Rc and -Rb1, respectively, from the upper spot.

A: TLC profile stained by sulfuric acid. B: Eastern blotting by anti-ginsenoside-Rb1 and anti-ginseside-Rg1 monoclonal antibodies I, II, III, IV, V and VI indicated white ginseng, red ginseng, fibrous ginseng (*P. ginseng*), *P. notoginseng*, *P. quinquefolius* and *P. japonicus*, respectively. Upper purple color spots and lower blue color spots were stained by anti-ginsenoside-Rg1 and anti-ginsenoside-Rb1 monoclonal antibodies, respectively.

The phloem contained higher concentration of ginsenoside Rb1 than xylem and cork.

The column was washed by phosphate buffer and then eluted by HOAc buffer containing KSCN and MeOH. Individual fractions (2 mL) were assayed by ELISA using anti-ginsenoside-Rb1 Mab.

The column was washed by phosphate buffer and then eluted by HOAc buffer containing KSCN and MeOH. Individual fractions (2 mL) were assayed by Eastern blotting using anti-ginsenoside-Rb1 Mab. Spots 1 and 2 were identified as chikusetsusaponin III and chikusetsusaponin III-20-O-gentiobiose, respectively, by comparison with authentic samples.

Lines 1,2 and 3 indicate crude extract, knock-out extract and purified ginsenoside-Rb1, respectively. Red spot shows ginsenoside-Rb1.
